# Development of novel polymeric nanoagents and their potential in cancer diagnosis and therapy runing title: Polymeric nanoagents for cancer theranostics

**DOI:** 10.3389/fchem.2022.1097205

**Published:** 2022-12-16

**Authors:** Ge Huang, Qian Li, Longyan Li, E. Wang

**Affiliations:** Department of Anesthesiology, Xiangya Hospital of Central South University, Changsha, China

**Keywords:** polymeric nanoagents, nanotechnologies, nanoparticles, cancer diagnosis, cancer therapy

## Abstract

Cancer has been one of the leading factors of death around the world. Cancer patients usually have low 5-year survival rates and poor life quality requiring substantial improvement. In clinic, the presenting diagnostic strategies lack sensitivity with only a small proportion of patients can be accurately identified. For diagnosed patients, most of them are at the advanced stages thus being delayed to receive treatment. Therefore, it is eager to investigate and develop highly effective and accurate techniques for cancer early diagnosis and individualized therapy. Various nanoplatforms are emerging as imaging agents and drug carriers for cancer theranostics recently. Novel polymeric nanoagents, as a potent exemplar, have extraordinary merits, such as good stability, high biosafety and high drug loading efficacy, showing the great prospect for cancer early diagnosis and precise treatment. Herein, we review the recent advances in novel polymeric nanoagents and elucidate their synthesis procedures. We further introduce the applications of novel polymeric nanoagents in cancer diagnosis, treatment, and theranostics, as well as associated challenges and prospects in this field.

## 1 Introduction

Cancer has been threatening human life and healthy, as a predominant driver to cause high morbidity and mortality worldwide (de Oliveira et al., 2020; Temkin et al., 2022). In the past few decades, substantial efforts have been devoted in cancer research, but the diagnosis and prognosis have not been improved much (de Oliveira et al., 2020). The main challenge behind is to identify the cancer patients in their early stage, so as to start personalized treatment in time.

Most cancer patients are diagnosed at the advanced stages because of lacking typical clinical symptoms. Conventional methods for cancer diagnosis mainly contain biopsy, magnetic resonance imaging (MRI), computed tomography (CT), positron emission tomography (PET), single photon emission computed tomography (SPECT) and ultrasound (US) (Solnik et al., 2022). Biopsy is still the gold standard, PET has a low resolution, and MRI causes a high false positive signal (Jin et al., 2021; Kowalchuk et al., 2021). Traditional cancer therapies, chemotherapy, radiotherapy, immunotherapy, and surgery face great challenges, such as low permeability, defective specificity, severe systemic side effects, and drug resistance (Liu et al., 2022a; Yazbeck et al., 2022). Therefore, it is imperative to investigate more effective therapeutic strategies for fighting against cancer.

Nanoagents have attracted great attention due to the easy-modified hydrophobic segments and rich functional groups, which are designed with the assistance of nanotechnologies and nanoparticles (NPs) (Huang et al., 2017). NPs dominantly comprise metal and metal oxide-based NPs, liposomes, dendrimers, magnetic NPs, quantum dots, and polymeric NPs (Selmani et al., 2022). Especially, Polymeric nanoagents, as polymeric NPs-assembled nanoagents, have made great progress for cancer diagnosis and treatment (Wang et al., 2020b; Yuan et al., 2021). Because of the high surface to volume ratio and the nanoscale size of NPs, polymeric nanoagents are capable of navigating through microvasculatures and across various biological barriers (Yuan et al., 2021). Hence, polymeric nanoagents can rapidly accumulate in cancer cells with the enhanced permeability and retention (EPR) effect as well as regulating the systemic biodistribution of therapeutic medicines (Wang et al., 2020b; Yuan et al., 2021).

Since the easy-modified hydrophobic segments and functional groups of nanoagents, lots of innovative polymeric nanoagents are developed by simultaneously conjugating with imaging agents and therapeutic molecules, such as fluorescent dyes, photosensitisers, aptamers, peptides, antibodies, chemotherapeutic drugs, or other biological molecules (Wang et al., 2020b; Haider et al., 2020; Yuan et al., 2021). Most importantly, growing preclinical trials have proved the increased biosafety, high selectivity, reduced systemic side effects, better solubility and stability of novel polymeric nanoagents (Li et al., 2022). These features have allowed polymeric nanoagents to perform specific imaging and precise therapy of cancer tissues, further developing into personalized polymeric theranostic nanoplatforms. Compared to conventional imaging methods, novel polymeric nanoagents-based imaging has a high temporospatial resolution, and numerous polymeric nanoagents have been developed for the early detection of cancer, such as indocyanine green (ICG) (Lv et al., 2022), lanthanide ion neodymium (Nd) (Deng et al., 2022), and chlorin e6 (Ce6)-based nanoagents (Odda et al., 2020). Recently, emerging methods for cancer treatment include novel polymeric nanoagents-based chemotherapy, gene therapy, photothermal therapy (PTT), and photodynamic therapy (PDT). PTT triggers irreversible pyroptosis, apoptosis, and necrosis of cancer cells *via* converting near-infrared (NIR) light into heat and further triggering hyperthermia (Yu et al., 2022). PDT stimulates cancer cells death through absorbing light and producing reactive oxygen species (ROS), especially singlet oxygen (Wang et al., 2022c). These polymeric nanoagents-based theranostic approaches are characterized with non-invasiveness, increased specificity, and low off-target toxicity (Wang et al., 2020a). Hence, this review would introduce the design of novel polymeric nanoagents, and their promising applications for the early diagnosis, treatment, and combined theranostics of cancer. Also, the unsolved problems of novel polymeric nanoagents in the field of oncology would be discussed.

## 2 The design and structure of novel polymeric nanoagents

Polymeric NPs lay a solid foundation for the development of novel polymeric nanoagents. Polymeric NPs originate from natural polymers and synthetic polymers in a core-shell structure, with hydrophilic blocks forming the shell and hydrophobic blocks forming the core of the NPs (Crucho and Barros, 2017). Generally, the synthesis of polymeric NPs is prepared by the two groups of methods, including preformed polymers and monomers polymerization-based encapsulating polymers (Crucho and Barros, 2017). Since the limitations of polymerization techniques, preformed polymers are more extensively utilized, where organic solvents are applied for dissolving the polymer in the first step (Zielinska et al., 2020). The preparation methods are composed of two-step and one-step procedures. Two-step procedures involving emulsification preparation and nanoparticles formation, mainly include emulsification-solvent evaporation, emulsification-solvent diffusion, and emulsification–reverse salting-out. One-step procedures consist of nanoprecipitation, dialysis, and supercritical fluid technology without emulsification to form nanoparticles (Crucho and Barros, 2017; Zielinska et al., 2020). In solvent evaporation method, the oil-in-water (o/w) emulsion is required for producing nanospheres. Emulsification diffusion requires the formation of an o/w emulsion that consists of a partially hydro-miscible organic solvent in the internal phase. The salting-out method separates a hydro-miscible solvent from an aqueous solution, and salting-out effect may facilitate the formations of nanospheres. Nanoprecipitation is considered as solvent displacement, depending on the interfacial deposition of a polymer after the organic solvent displacing from a lipophilic solution to the aqueous phase. During the synthesis of polymeric NPs, multiple impurities can exist in NPs suspension or adsorb into the surface of NPs, including organic solvents, salts, and particle residues. These toxic impurities must be removed with filtration, centrifugation, and dialysis techniques (Drozdz et al., 2020). Taken together, it is essential for choosing the preferable preparation method based on the drug’s characteristics and the desired properties of polymeric NPs.

**FIGURE 1:**
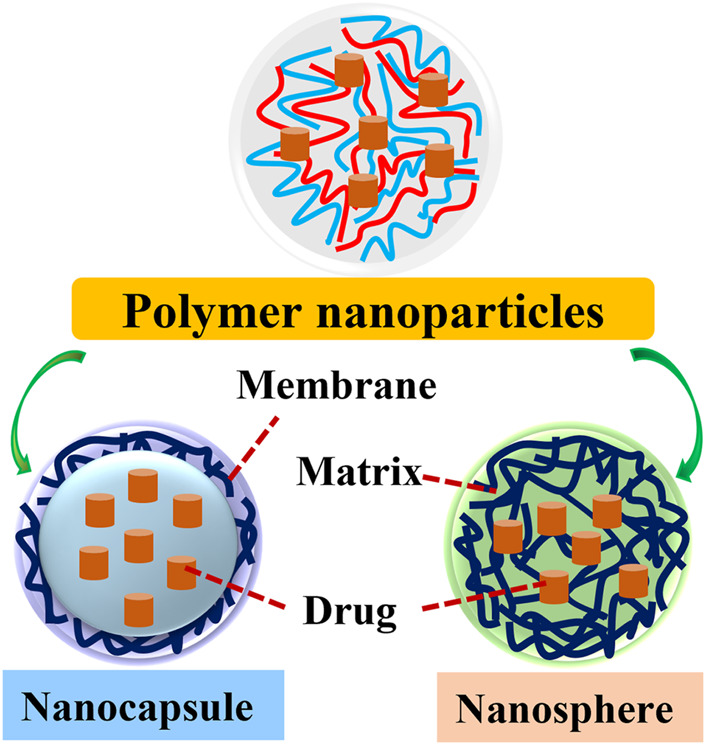
The structure of two types of polymer nanoparticles. Polymer NPs are classified into nanocapsules and nanospheres. Polymeric nanosphere is a matrix type, insoluble solid-colloidal particle in which drugs are distributed throughout the chemical compound matrix. Polymeric nanocapsule is a colloidal-vesicular system where the drug is confined to a cavity surrounded by a distinctive compound membrane.

According to the morphology of polymeric NPs, they are classified into nanocapsules and nanospheres. Polymeric nanosphere is an insoluble solid-colloidal particle in which drugs are dissolved, entrapped, encapsulated, chemically bound or adsorbed to the constituent polymer matrix. Polymeric nanocapsule is a colloidal vesicle where drugs are confined to an oily reservoir or within an aqueous cavity surrounded by the polymer membrane (Crucho and Barros, 2017) (Naseef, P.P et al., 2021) (Figure 1). The special core-shell structure allows the selective delivery of drugs or fluorescent molecules to cancer tissues. Then, the release of drugs is achieved by modulating the rates of polymer biodegradation and drugs diffusion in the polymer matrix as well as induced by exogenous and endogenous stimuli in the specific disease microenvironments (Kamaly et al., 2016; Singh et al., 2021). Ultimately, the polymer matrix is degraded into non-hazardous molecules and excreted from the body.

**FIGURE 2:**
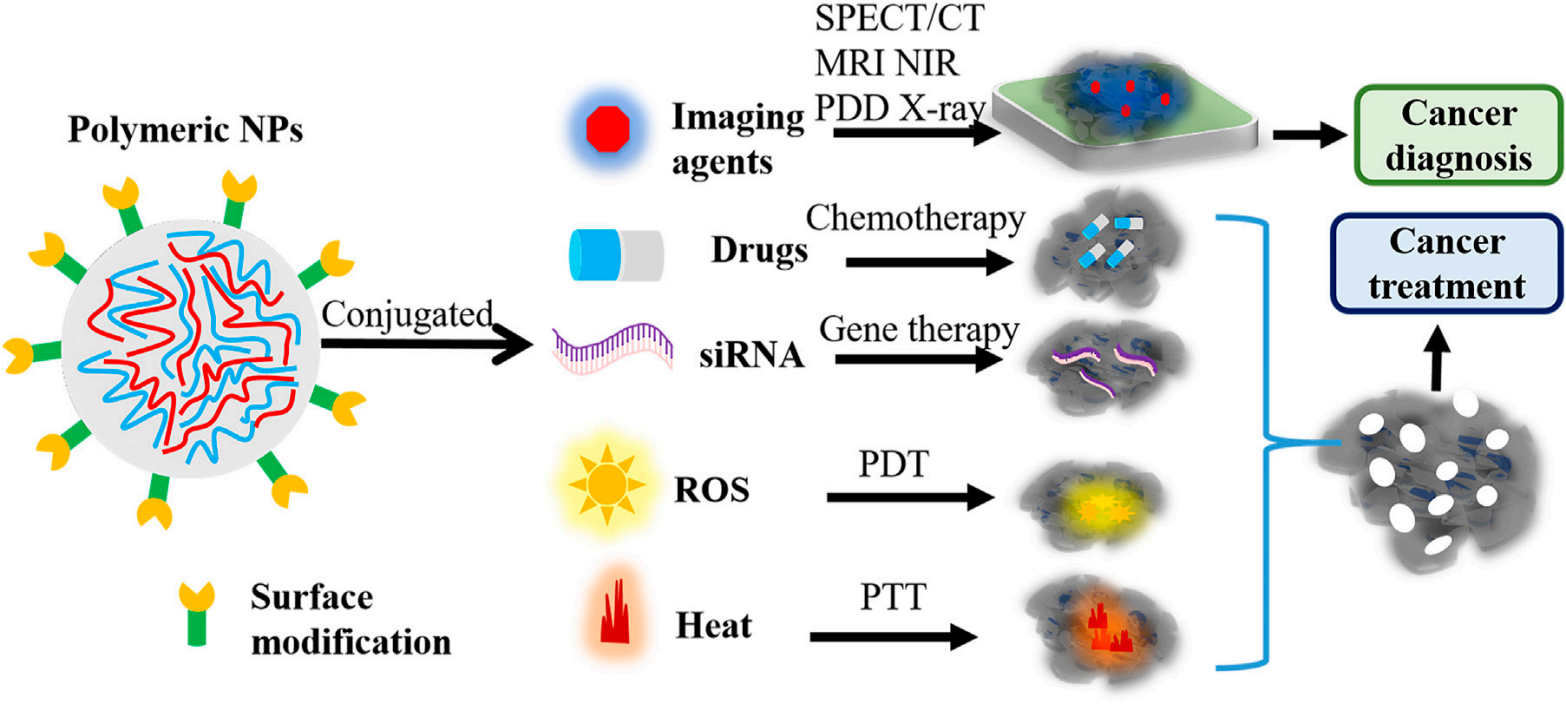
The function of polymeric nanoagents in cancer diagnosis and treatment. Polymeric NPs-based nanoagents would be widely used to guide SPECT/CT, MRI, NIR, PDD, and X-ray imaging through integrating with imaging agents. Also, these novel polymeric nanoagents can guide chemotherapy, siRNA therapy, PTT and PDT through integrating with chemotherapeutic drugs, siRNA, and imaging agents. NPs, nanoparticles; PDD, photodynamic diagnosis; NIR, near-infrared; PTT, photothermal therapy; PDT, photodynamic therapy; NPs, nanoparticles; ROS, reactive oxygen species; MRI, magnetic resonance imaging; CT, computed tomography; SPECT, single photon emission computed tomography.

Polymeric NPs-based nanoagents are characterized by effective drug-polymer interactions and can inhibit the premature release of drugs (Villamizar-Sarmiento et al., 2021). Moreover, they exhibit non-toxicity, good water-solubility, extensive biocompatibility, and easy modification (Crucho and Barros, 2017; Villamizar-Sarmiento et al., 2021). In virtue of these unique features, novel polymeric nanoagents have served as prospective candidates for cancer precise diagnosis and therapy *via* guiding MRI, SPECT/CT, NIR, X-ray imaging, photodynamic diagnosis (PDD), PTT, and PDT after conjugating with imaging agents and therapeutic drugs (Figure 2).

## 3 The applications of novel polymeric nanoagents in cancer diagnosis

In clinic, a number of cancer patients suffer from metastasis and advanced cancer stages due to the delayed diagnosis. Increasing evidence has shown that the early detection of cancer can significantly decrease mortality rate and improve therapeutic efficacy (Ribeiro et al., 2022; Zhu et al., 2022). However, conventional imaging techniques and biomarkers detection are not sufficient for the early diagnosis. Fortunately, polymeric nanoagents-based accurate bioimaging can detect small tumors at the early stage as well as reveal the biological processes involved in early carcinogenesis after conjugating with non-invasive optical imaging agents (Casas, 2020). Herein, novel polymeric nanoagents would be extensively utilized for cancer diagnosis due to their high sensitivity, non-invasiveness and good biocompatibility, and they will greatly advance the field of oncology (Shanavas et al., 2017; Tiwari et al., 2017) (Table 1).

**TABLE 1:** The applications of novel polymeric nanoagents in cancer diagnosis.

Types	Conjugation	Imaging techniques	Indications	Advantages	Ref
Fol-cht-based polymer	Technetium-99m	SPECT/CT	Folate receptor-overexpressed cancers	Targeting to folate receptor-overexpressed cancer cells	Polyak et al. (2014)
Fol-cht-based polymer	Magnetic PLGA	MRI	Oral cancer cells	Providing SPIONs delivery, further shortening T2 relaxation time and enhancing nanoparticle relaxivity	Shanavas et al. (2017)
Fol-cht-based polymer	5-ALA	Performing 5-ALA-based PDD	Oral cancer, CIN, prostate cancer, glioblastoma	Increasing the uptake of 5-ALA, the accumulation of PpIX fluorescence and facilitating the photodynamic detection	Yang et al. (2013); Babic et al. (2018); Casas, (2020); Xu et al. (2021)
SPNs	TPP	NIR	Various cancers	Producing shifted NIR luminescence, allowing the *in vivo* tiny cancer imaging *via* sensing low oxygen	Cui et al. (2018)
SPNs	AF	PA	Various cancers	Generating stronger PA signal and improving photodiagnostic efficacy	Li et al. (2018)
SPNs	RBC	PA	Various cancers	Enhancing NIR light absorption and photostability	Zheng et al. (2020)
HA	ICG	NIR	Prostate cancer	Augmenting optical stability and reducing systemic toxicity	Souchek et al. (2018)
BSA	ICG	NIR-I	Cervical cancer, neuroblastoma	Producing strong NIR-I fluorescence emission	Ma et al. (2021); Clutter et al. (2022)
PSMA	ICG	NIR	Cervical cancer	Improving the chemical stability and biocompatibility	Chen et al. (2021)
PLGA	ICG	NIR	Cervical cancer	Enhancing the NIR stability and internalization, reducing cytotoxicity	Choi et al. (2021)
PEG	ICG	NIR	Various cancers	Showing more sensitive imaging and strong targeting ability	Wang et al. (2022b)
PNMs	ICG	NIR	Various cancers	Inhibiting ICG leakage and indicating favorable biocompatibility	Hsu et al. (2020)
PAH	ICG	NIR	Ovarian cancer	Enhancing the fluorescence stability and targeting ability	Bahmani et al. (2014)
PCL	ICG	NIR, X-ray	Various cancers	Boosting the fluorescence stability, and having minimized adverse effects on the surrounding tissues	Gorecka et al. (2022)

Chitosan is a biocompatible, biodegradable, and minimized invasive polymer, and it has the widespread application for cells optical imaging. It is demonstrated that most epithelial cancer cells highly express folate receptor, and folate-chitosan (fol-cht)-based polymeric nanoagents can be applied for SPECT/CT imaging to detect folate receptor-overexpressed cancer tissues after radiolabeling with technetium-99m (Polyak et al., 2014). In addition, a study synthesizes hybrid nanoagents with a magnetic poly (lactide-co-glycolide) (PLGA) core and a fol-cht-based shell, and the shell surface’s hydroxyl (-OH) and amine (-NH_2_) functional groups can be easily modified through complicated chemical reactions. The biocompatible hybrid nanoagents provide super paramagnetic iron oxide NPs (SPIONs) delivery for targeting to folate receptor-overexpressed oral cancer cells, further bettering MRI contrast with the shortened T2 relaxation time and enhanced nanoparticle relaxivity (Shanavas et al., 2017). Taken together, chitosan shell-based polymeric nanoagents have showed a prospective potential for the early diagnosis of various cancers with better imaging contrast.

5-aminolaevulinic acid (5-ALA) is a hydrophilic and zwitterionic imaging drug as well as a precursor of heme and chlorophyll (Harada et al., 2022). Exogenous administration of 5-ALA ultimately metabolizes into protoporphyrin IX (PpIX), and accumulated PpIX in cancer cells generates PpIX fluorescence, further performing 5-ALA-based PDD (Beika et al., 2021; Harada et al., 2022). Nevertheless, 5-ALA shows a poor affinity toward the cell membrane, and cancer cells are difficult to take up 5-ALA (Yang et al., 2013). Considering the folic acid receptor-mediated endocytosis promoting the uptake of 5-ALA, 5-ALA is conjugated with succinate-modified chitosan (SCHI) and fol-cht-based polymer NPs (Casas, 2020). Once taken up by cancer cells, 5-ALA releases from lysosome due to the wakened attraction intensity between chitosan and 5-ALA, which strengthens the accumulation of PpIX fluorescence and facilitates the PDD of cancer cells (Yang et al., 2013). Numerous research have indicated the great prospect of the folic acid-based polymeric nanoagents for diagnosing cancer by delivering 5-ALA, such as oral cancer (Yang et al., 2013), cervical intraepithelial neoplasia (CIN) (Xu et al., 2021), prostate cancer (Casas, 2020) and glioblastoma (Babic et al., 2018).

Semiconducting polymer nanoparticles (SPNs) are also identified as excellent optical agents and applied for fluorescence, chemiluminescent, and photoacoustic (PA) imaging for the early detection of cancer due to the excellent biocompatibility and structural versatility (Cui et al., 2020; Tang et al., 2022). Poly (p-phenylenevinylene) (PPV)-based SPNs could emit afterglow luminescence after removing light excitation. Hence, the tetraphenylporphyrin (TPP) photosensitizer-copolymerized PPVs SPNs are synthesized to function as near-infrared (NIR) afterglow nanoagents. The novel copolymer nanoagents produce red-shifted NIR luminescence and amplified afterglow signals, allowing the *in vivo* tiny cancer imaging *via* sensing low oxygen in the cancer microenvironment (Cui et al., 2018). In general, cancer-associated fibroblasts are recognized as the key barriers for cancer therapy. Thereby, activated fibroblasts (AF)-camouflaged SPNs (AF-SPNs) are emerged as effective biomimetic nanoagents for optimizing cancer phototheranostics, where a SPN and AF membrane serve as the core and shell, respectively (Li et al., 2018). The AF-SPNs would generate stronger PA signal and dramatically strengthen photodiagnostic efficacy (Li et al., 2018). As well, RBC membrane-coated SPNs have the enhanced NIR light absorption and photostability for PA imaging (Zheng et al., 2020), which can deeply penetrate into cancer tissues and be rapidly cleared due to the small size. Both RBC and AF membrane-modified polymeric nanoagents provide the remarkable PA imaging contrasts for diagnosing cancer tissues (Li et al., 2018; Zheng et al., 2020).

In clinic, ICG is a usually used NIR fluorescence contrast agent. But the poor optical stability and clearance efficacy of ICG limit its applications. To overcome these demerits, ICG is encapsulated into hyaluronic acid (HA) for NIR imaging, HA is a natural and biodegradable polysaccharide polymer (Souchek et al., 2018). The ICG-lorded HA nanoagents have showed the prominent value in detecting prostate cancer and CD44-positive cervical cancer (Souchek et al., 2018). Bovine serum salbumin (BSA) is another common natural polymer, and BSA-coated ICG nanoagents exhibit non-toxicity, good water-solubility and strong NIR-I fluorescence emission for cancer imaging, such as cervical cancer (Ma et al., 2021) and neuroblastoma (Clutter et al., 2022). Besides natural polymers, a study encapsulates ICG with synthetic poly (styrene-co-maleic anhydride) (PSMA) to construct ICG@PSMA nanoagents. Poly PSMA is an amphiphilic polymer that can encapsulate ICG to improve its chemical stability and biocompatibility. Further, ICG@PSMA nanoagents exhibit strong NIR fluorescence as well as reduced systemic toxicity for cervical cancer cells (Chen et al., 2021). Also, ICG can be encapsulated into other synthetic polymers to perform NIR imaging, including PLGA (Choi et al., 2021), polyethylene glycol (PEG) (Wang et al., 2022b), polymeric nanomicelles (PNMs) (Hsu et al., 2020), poly (allylamine hydrochloride) (PAH) (Bahmani et al., 2014), poly caprolactone (PCL) (Chien et al., 2018; Gorecka et al., 2022).

## 4 The role of novel polymeric nanoagents in cancer treatment

The progression of cancer is a relatively complex process, involving in cellular and genetic alterations. Although, advancements in cancer therapy have improved the survival rates and reduced the deaths, there are undesirable side effects. In recent, polymeric nanoagents-guided chemotherapy, nucleic acid therapy, PDT and PTT, emerge as remarkable approaches for achieving cancer treatment (Wang et al., 2022c; Yu et al., 2022) (Table 2). These advanced strategies selectively target into cancer tissues with minimal invasion into healthy tissues as well as prominently enhance the therapeutic efficacy (Liu et al., 2022b; Macchi et al., 2022).

**TABLE 2:** The role of novel polymeric nanoagents in cancer treatment.

Types	Conjugation	Treatments	Indications	Advantages	Ref
ACC-SF	DOX	Chemotherapy	Various cancers	Blocking the premature efflux of DOX and preventing the protonation of DOX within the lysosome, displaying the excellent therapeutic performance	Tan et al. (2019)
LPNPs	Sal	Chemotherapy	Pancreatic cancer	Increasing oral absorption and cancer cells uptake	Fang et al. (2014)
Polymeric micelles	PTX	Chemotherapy	Breast cancer	Enhancing water-solubility, bioavailability, and reducing toxicity	Wang et al. (2017)
Triacontanol polymer	DTX	Chemotherapy	Pancreatic cancer, breast cancer, prostate cancer	Improving water-solubility and oral bioavailability, further enhancing the cancer cells-killing effects	Almawash et al. (2018); Lu et al. (2020); Xiao et al. (2022)
GC-based polymer	PTX, cisplatin, CPT, DTX	Chemotherapy	Various cancers	Resulting in maximized therapeutic efficacies	Hao et al. (2019); Dutta et al. (2021); Ren et al. (2021); Wang et al. (2021)
GC-based polymer	C60	PDT	Various cancers	Generating ROS and guiding PDT	(Kim et al. (2014); Gunduz et al. (2022)
PSMA NPs	ICG	PTT	Cervical cancer	Augmenting PTT efficiency under 808 nm laser irradiation	Chen et al. (2021)
SPNs	AF	PTT, PDT	Various cancers	Releasing single oxygen and heat for strengthening PDT and PTT	Li et al. (2018)
SPNs	RBC	PTT	Various cancers	Exert dramatic photothermal conversion and photothermal killing efficacy against cancer cells	Zheng et al. (2020)
PEG-PCL	C3, ICG	PDT, PTT	OSCC	Combining PTT and PDT treatment with better photothermal conversion stability	Ren et al. (2017)
HSA	Cisplatin, ICG	Chemotherapy, PTT, PDT	Various cancers	Displaying the synergistic anti-cancer effects of PDT, PTT and chemotherapy	Wang et al. (2019b)
GC-PEI	RFP-siRNA	Gene therapy	Various cancers	Delivering siRNA to the cell cytoplasm and exerting remarkable silencing effects	Huh et al. (2010)
tGC polymer	VEGF-siRNA	Gene therapy	Various cancers	Achieving VEGF knockdown and performing anti-cancer effects	Kim et al. (2017)
PEG-PEI-Ce6	Wnt1-siRNA	PDT, Gene therapy	Oral cancer	Hindering EMT process and enhancing the killing effects	Ma et al. (2017)
Polymeric micelles	PTX	Gene therapy, chemotherapy	Various cancers	Co-Delivering PTX and siRNA, and boosting the synergistic anti-cancer effects	Shi et al. (2021)

It is worth noting that premature drug leakage, low drug delivery efficiency and defective cellular uptake are considerable barriers for cancer chemotherapy, and assembling chemotherapeutic drugs with polymer NPs might solve these problems. Herein, the tumor microenvironment (TME)-responsive biocompatible nanoagents are constructed using amorphous calcium carbonate (ACC) cores and silk fibroin (SF) shells. Upon entering into TME, SF shells concurrently inhibit premature drug release and target to the acidic lysosomes. And the sensitive ACC NPs are gradually degraded, further producing a majority of Ca and CO and resulting in lysosomal collapse. Doxycycline (DOX) is a widely used anti-cancer drug. ACC-SF NPs-conjugated DOX nanoagents block the premature efflux of DOX from cancer cells and prevent the protonation of DOX within the lysosome, displaying the excellent therapeutic performance (Tan et al., 2019). Salidroside (Sal) is a potent anti-cancer drug with high water-solubility. The clinic application of Sal in cancer therapy has been restricted by poor oral absorption and low cancer cells uptake. To overcome this impediment, Sal is lorded with lipid-shell and PLGA-PEG-PLGA triblock polymer-core NPs (Sal-LPNPs) by a double emulsification method (Fang et al., 2014). Sal-LPNPs nanoagents have a distinct inhibitive effect on the growth of human pancreatic cancer cells, but its clinical use needs more profound exploration. Paclitaxel (PTX) is an effective anti-cancer drug for various solid cancers, but it has low solubility, poor bioavailability, and inevitable toxicity. Encapsulation of PTX in polymeric micelles can boost its water-solubility and bioavailability, which has the promising applications in breast cancer therapy (Wang et al., 2017). Docetaxel (DTX) is a taxane-based anti-cancer drug with low water-solubility and oral bioavailability. Encapsulating DTX with PLGA, liposomes and PEGylated triacontanol polymer systems can dramatically enhance the DTX delivery efficacy and cancer cells-killing effects, like pancreatic cancer (Almawash et al., 2018), breast cancer (Lu et al., 2020), and prostate cancer (Xiao et al., 2022). In addtion, glycol chitosan (GC)-based polymeric nanoagents have been widely used for delivering chemotherapeutic drugs *via* hydrophobic interactions, such as paclitaxel (Hao et al., 2019), cisplatin (Wang et al., 2021), camptothecin (CPT) (Dutta et al., 2021), or DTX (Ren et al., 2021), resulting in maximized therapeutic efficacies.

Besides chemotherapy, PDT-produced ROS and PTT-generated heating specifically trigger phototoxic death of cancer cells with the assistance of optical nanoagents (Liu et al., 2022b; Macchi et al., 2022). Fullerene (C60) possess remarkable photophysical properties, thereby it can be used as a potentially strong photoactivatable agent for PDT *via* triggering ROS production (Gunduz et al., 2022). In detail, C60 is conjugated with GC to form GC-C60 or GC-2,3-dimethylmaleic acid (DMA)-C60, the solubilized C60 nanoagents produce tremendous fluorescence signals, further accumulating in various cancer tissues to guide PDT (Kim et al., 2014). Also, ICG@PSMA shows good PTT efficiency in cervical cancer cells under 808 nm laser irradiation. Therefore, ICG@PSMA nanoagents might serve as photothermal nanoagents (PTN) for other different types of cancer treatment, providing a solid basis for following *in vivo* experiments (Chen et al., 2021). Additionally, GC-based polymeric nanoagents are preferable carriers for PTT and PDT (Rhee et al., 2014).

SPNs is stand out as superb optical agents for PDT and PTT, and cell membrane-coated SPNs have indicated superior therapeutic effects (Tang et al., 2022). For instance, the AF-SPNs nanoagents generate not only NIR fluorescence and PA signals for imaging but also single oxygen and heat for strengthening photodynamic and photothermal therapeutic efficacies *via* promoting NPs accumulation in cancer cells (Li et al., 2018). As well, RBC membrane-coated SPNs have excellent photothermal conversion efficiency for PTT. These data demonstrate that RBC membrane-coated SPNs exert dramatic photothermal killing efficacy against cancer cells, which would become a promising phototheranostic agent for clinical translation (Zheng et al., 2020).

In particular, recent studies have proved that the combined treatment of chemotherapy, PTT and PDT has the stronger therapeutic effects. For instance, ICG is conjugated with organic compound (C3) and polyethylene glycol-polycaprolactone (PEG-PCL) to construct hybrid nanoagents (PEG-PCL-C3-ICG) for combined PTT and PDT treatment. *In vitro* and *in vivo* experiments of oral squamous cell carcinoma (OSCC) cells show that PEG-PCL-C3-ICG nanoagents have better performance than PTT or PDT separately, together with better photothermal conversion stability, lower cytotoxicity, and faster metabolic rate (Ren et al., 2017). In line with these, human serum albumin indocyanine green-cisplatin nanoagents (HSA-ICG-DDP) are designed to combine PDT, PTT with chemotherapy. *In vitro* and *in vivo* experiments have demonstrated that the synergistic anti-cancer effects of PDT, PTT and chemotherapy are remarkably heightened compared to ICG, HSA-ICG and DDP treatments, showing the favorable value of polymeric nanoagents in combination therapy (Wang et al., 2019b).

Small interfering RNA (siRNA), as one of nucleic acid therapy, is an effective therapeutic agent due to its specific gene silencing ability. However, the application of siRNA is hindered due to its susceptibility to nuclease degradation and low internalization by cancer cells. To develop novel delivery systems of siRNA, GC-PEI NPs are assembled by combining GC-5β-cholanic acid and polyethylenimine (PEI) polymers-5β-cholanic acid at a 1:1 weight ratio, then mixing RFP-siRNA and GC-PEI NPs at a 1:5 weight ratio (Huh et al., 2010). The generated GC-PEI NPs can effectively deliver siRNA to the cell cytoplasm and exert remarkable silencing effects, which are verified in RFP-expressing B16F10 cells (Huh et al., 2010). Besides, the thiolated GC (tGC)-polymerized siRNA is developed to enhance the stability of the siRNA (Kim et al., 2017). *In vivo* fluorescence imaging results reveal that VEGF-siRNA-tGC nanoagents have the increased serum stability, and they can quickly internalize and localize into the cytosol, further achieving VEGF knockdown and performing anti-cancer effects (Kim et al., 2017).

Most importantly, siRNA represents the enhanced synergistic anti-cancer effects when combining with chemotherapeutic drugs or PDT. For instance, siRNA Wnt1 is introduced into polyethylene glycol-polyethyleneimine-chlorin e6 (PEG-PEI-Ce6) nanoagents. Further, siRNA Wnt1-PEG-PEI-Ce6 nanoagents target into the cytoplasm of PDT-treated oral cancer cells to hinder EMT process and boost the killing effects against cancer cells (Ma et al., 2017). For another thing, siRNA integrated with PTX is co-delivered by pH-sensitive polymeric micelles, which can not only achieve gene silencing but also block premature drug release and perform chemotherapeutic effects (Shi et al., 2021). These combined treatments bring great advancements in nanotechnology, nanomedicine, drug delivery, and cancer therapy.

## 5 The role of novel polymeric nanoagents in cancer theranostics

In order to simultaneously deliver therapeutic drugs and diagnostic imaging agents as well as real-timely monitor of therapeutic responses, imaging-guided theranostic nanoagents are developed (Hu et al., 2021a) (Table 3). The strategy of treatment with simultaneous visual diagnosis benefits the formulation of individualized therapy planning and the development of precise medicine. Nevertheless, it is required for figuring out the whole-body 3D information and the dynamic biological processes of theranostic nanoagents for *in vivo* applications, including absorption, distribution, metabolism, and excretion.

**TABLE 3:** The role of novel polymeric nanoagents in cancer theranostics.

Types	Conjugation	Theranostics	Indications	Advantages	Ref
Cy@Silk	Tc	SPECT and real-timely monitoring	Various cancers	Visualizing the distribution of nanoagents, real-timely monitoring cancer progression and maximizing the therapeutic efficacy	Wang et al. (2019a)
SPNs	Peroxidase	NIR-II-based PA and PTT	Various cancers	Elevating the sensitivity of diagnosis and the efficacy of PTT with the stronger PA signals	Lyu et al. (2018)
SPNs	—	NIR-II-based PA and PTT	Gliomas	Augmenting the absorption and inducing cancer cells death in both shallow and deep tissues	Wen et al. (2020)
AIE	ApoE	NIR-II-based PTT	GBM	Showing a higher PTT efficiency	Wang et al. (2022a)

Presently, the NIR cypate-induced SF self-assembly (Cy@Silk) nanoagents are designed and labeled with the radionuclides (Tc) for SPECT imaging. The multimodal SPECT imaging can offer the whole-body 3D information about nanoagents' distribution *in vivo*, substantially facilitating the real-timely monitoring of cancer progression and maximizing the therapeutic efficacy (Wang et al., 2019a). Besides, biodegradable SPNs are developed with the ability to augment PA imaging and PTT efficacy. The biodegradable SPNs are designed based on the enzymatically oxidizable nature of vinylene bonds, and they can be transformed into water-soluble nanoparticles (SPNV). The vinylene bonds within the polymer backbone endow SPNV with an excellent mass absorption coefficient (1.3-fold) and a photothermal conversion efficacy (2.4-fold). Hence, SPNV shows the stronger PA signals and higher photothermal maximum temperature, dramatically elevating the sensitivity of diagnosis and the efficacy of PTT for various cancers (Lyu et al., 2018).

Considering the restricted absorption of the first NIR window (NIR-I) nanoagents, SPNs under the second NIR window (NIR-II) are assembled to augment the absorption. In light of the excellent PA and photothermal performance, high photostability, proper size, and low toxicity of SPNs, NIR-II-based SPNs nanoagents are assimilated by U87 glioma cells. Then, they lead to efficient cells death under NIR-II light irradiation, allowing PA imaging and PTT toward gliomas in both shallow and deep tissues (Wen et al., 2020). In another study, brain-targeted NIR-IIb aggregation-induced-emission (AIE) nanoagents are synthesized to graft apolipoprotein E peptide (ApoE), which is termed as ApoE-Ph nanoagents (Wang et al., 2022a). ApoE-Ph nanoagents have a higher PTT efficiency for glioblastoma (GBM) by keeping the balance of radiation-modulated NIR-fluorescence imaging at 1,550 nm and non-radiation NIR-PTT, opening a novel window for boosting theranostics in other cancers (Wang et al., 2022a). Also, nanoagents under NIR-II have the precise multimodel imaging capability and concurrent PTT, such as dual-model (NIR-II/MRI)-guided cancer theranostics (Hu et al., 2021b), and tri-modal (PA/NIR-II/MRI) imaging-guided PTT (Hu et al., 2019).

## 6 The challenges of novel polymeric nanoagents

Novel polymeric nanoagents have been investigated for a long time and provide new insights for the diagnosis and treatment of cancer, and some polymeric nanoagents are entering clinical trials. However, novel polymeric NPs also have several drawbacks requiring further attention. Neutral or negatively charged and larger nanoagents are prone to escape the immune system with the reduced EPR effects, and positively charged and smaller NPs are poorly excreted and have the potential toxicity (Liang et al., 2021). These properties of nanoagents might affect the diagnostic and therapeutic efficacy for cancer (Crucho and Barros, 2017). Thereby, the size, charge, shape, and hydrophilicity of polymeric nanoagents should be further optimized.

On the other hand, the change of TME (like temperature and pH) is an important issue that attenuates the effectiveness of polymeric nanoagents-based theranostic systems, and the corresponding mechanisms remain unclear. In order to timely adjust and optimize the polymeric nanoagents-based theranostic strategies, the advanced equipment can be used to monitor the change of TME. On the other side, an innovative wearable electronic strain sensor might be applied for timely assessing therapeutic response of cancer patients by discerning differences in tumor volume dynamics (Abramson et al., 2022). Another pressing drawback of nanoagents-based systems is the rapid initial or burst release, which is often attributed to the weakly bound to the surface (Yang et al., 2014). Hence, supramolecular chemistry, especially host-guest chemistry might markedly strengthen the interaction between drugs and polymer chains (Yang et al., 2014).

Thirdly, the relatively large dose of polymeric nanoagents is administrated *in vitro* mouse models, but showing a relatively short circulating lifetime. Further clinical trials should try to reduce the dose and extend lifetime *via* adjusting medication plan, modifying with human serum albumin (HSA), polysaccharides or PEG (Hu et al., 2018). Meanwhile, the large discrepancies between *in vivo* and *in vitro* experiments should also be concerned, thereby, the more homologous animals should be chosen for experiments.

Lastly, the manufacturing processes of theranostic nanosystems for simultaneous diagnosis and therapy are generally complicated, and the productivity of novel nanoagents needs to be improved. So, the technical challenges, such as cost, colloidal stability, and reproducibility, must be taken into account. Future experiments should be focused on investigate cost-effective nanomaterials and simply the productive procedures.

## 7 Conclusion

The advanced polymeric nanoagents have obtained increasing attention in oncology research and biomedicine, wherein nanoagents not only function as imaging agents but also as the delivery carriers of drugs. Most importantly, novel polymeric nanoagents-based theranostic systems have the great potential to achieve the simultaneous treatment and diagnosis of cancer; accordingly, there are future prospects to prolong the survival of cancer patients. In the coming years, more profound research would pay attention to optimizing the physicochemical properties of nanoagents, improving productivity, lowering the production costs, and rapidly translating polymeric nanoagents into clinical applications. Although there is a long way to go until clinical translation, novel innovative polymeric nanoagents offer a great opportunity for improving current strategies for early cancer detection, diagnosis and treatment procedures, and they will be welcome in the future.

## Glossary

Fol-cht,: folate-chitosan;
PLGA,: poly (lactide-co-glycolide);
PDD,: photodynamic diagnosis;
SPIONs,: super paramagnetic iron oxide NPs;
CIN,: cervical intraepithelial neoplasia;
5-ALA,: 5-aminolaevulinic acid;
PpIX,: protoporphyrin IX;
SPNs,: semiconducting polymer nanoparticles;
PA,: photoacoustic;
TPP,: tetraphenylporphyrin;
HA,: hyaluronic acid;
ICG,: indocyanine green;
BSA,: bovine serum salbumin;
PSMA,: poly (styrene-co-maleic anhydride);
PEG,: polyethylene glycol;
PNMs,: polymeric nanomicelles;
PAH,: poly (allylamine hydrochloride);
PCL,: poly caprolactone;
NIR,: near-infrared;
MRI,: magnetic resonance imaging;
CT,: computed tomography;
SPECT,: single photon emission computed tomography;
ACC,: amorphous calcium carbonate;
SF,: silk fibroin;
DOX,: doxycycline;
Sal,: salidroside;
LPNPs;: lipid-shell and PLGA-PEG-PLGA triblock polymer-core nanoparticles;
PTX;: paclitaxel;
DTX,: docetaxel;
GC,: glycol chitosan;
CPT,: camptothecin;
C60,: fullerene;
ICG,: indocyanine green;
PSMA,: poly (styrene-co-maleic anhydride);
SPNs,: semiconducting polymer nanoparticles;
AF,: activated fibroblasts;
C3,: organic compound;
OSCC,: oral squamous cell carcinoma;
HAS,: human serum albumin;
PEI,: polyethylenimine;
tGC,: thiolated GC;
Ce6,: chlorin e6;
siRNA,: small interfering RNA;
PEG,: polyethylene glycol;
PTT,: photothermal therapy;
PDT,: photodynamic therapy
